# A Case of Postherpetic Itch Effectively Treated With Pulsed Radiofrequency Therapy

**DOI:** 10.7759/cureus.75425

**Published:** 2024-12-09

**Authors:** Hiroki Nakamura, Ikuo Uekita, Keiji Hashizume

**Affiliations:** 1 Pain Center, Kouseikai Takai Hospital, Tenri, JPN

**Keywords:** fluoroscopy, herpes zoster, nerve block, neuralgia, pruritus, pulsed radiofrequency treatment

## Abstract

We report a case of a 65-year-old female with postherpetic itch (PHI) over the left chest, who experienced significant relief after pulsed radiofrequency (PRF) therapy. While her initial pain and rash had improved with nerve blocks and medications, she had developed severe itching. PRF therapy significantly reduced the itching, which nearly disappeared. This report suggests that neuropathic pain treatments, like PRF, may also be effective for PHI, a condition that is not fully understood and has limited treatment options but can severely impact the quality of life.

## Introduction

Herpes zoster (HZ) is sometimes associated with itching as well as persistent pain [1]. Postherpetic itch (PHI) is reported in approximately 28% of patients with postherpetic neuralgia (PHN) and can cause significant discomfort and disability [1,2]. Similar to PHN, there is a lack of standardized treatment for PHI, and the current approaches include medications, nerve blocks, and pulsed radiofrequency (PRF) therapy, with no definitive consensus [2,3,4]. PRF has been shown to be effective in treating neuropathic pain [5], and its use in PHI is supported by case reports [6]. However, the literature on it is limited. We report a case of PHI over the left chest symptomatically managed with PRF therapy, indicating its potential utility for this condition.

## Case presentation

**Figure 1:**
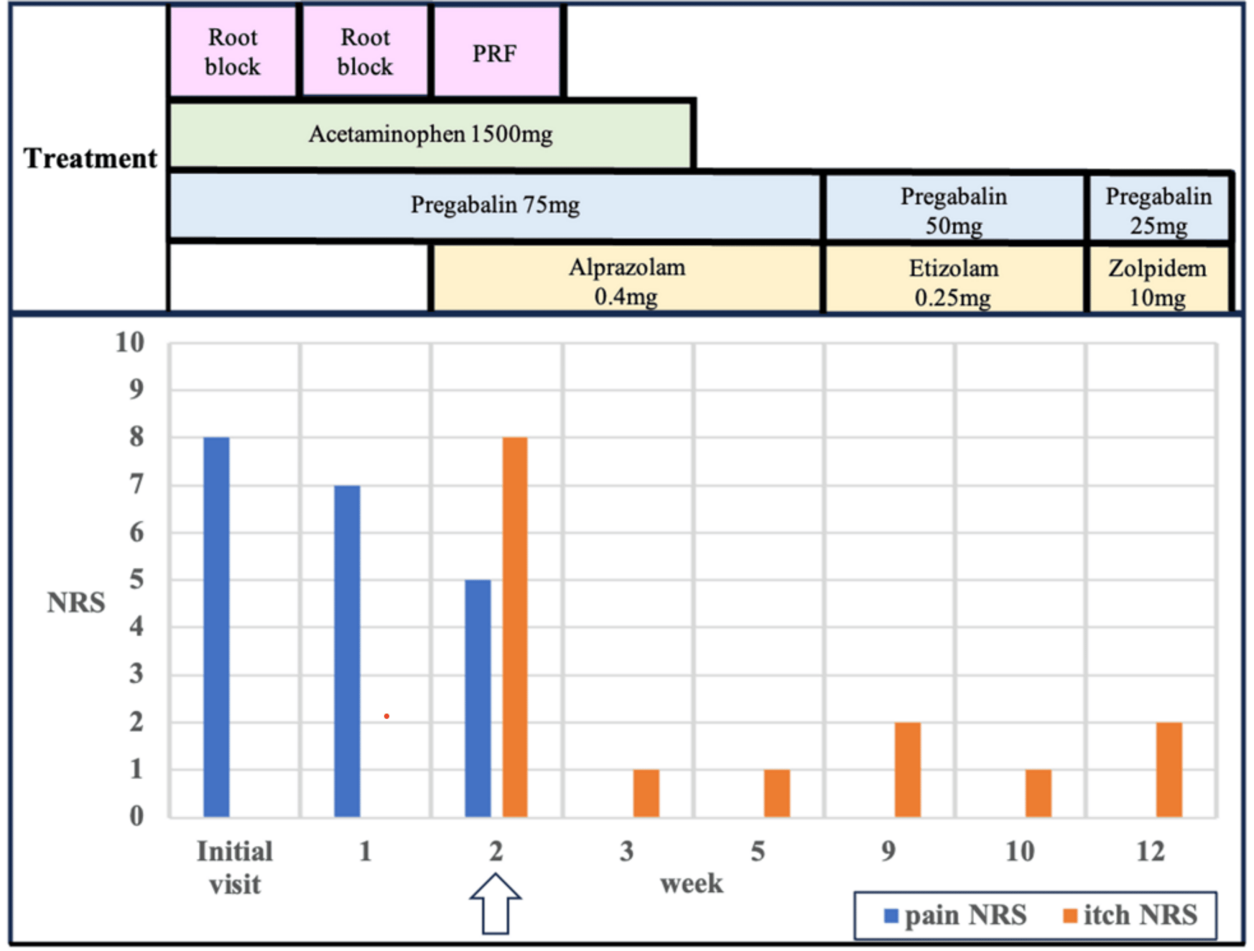
Overall course of treatment and change in the degree of itching The patient's rash had started 10 days before the initial visit. After two nerve blocks, we performed PRF therapy (white arrow). After PRF, both pain and itching NRS reduced to 1. And 120 days after the onset, she was symptom-free NRS: Numeric Rating Scale; PRF: pulsed radiofrequency

A 65-year-old female presented with tingling pain under her left breast, spreading to her back. She had developed a rash 10 days prior and had been diagnosed with HZ. Despite receiving loxoprofen, her pain had worsened, and she had been referred to our clinic with sharp, shooting pain [Numeric Rating Scale (NRS): 8/10] and dull, continuous pain (NRS: 6/10), along with allodynia and sleep disturbances. Physical examination revealed a rash extending from her left chest to her back, with reduced sensation on the left side. Suspecting HZ affecting the T6 nerve root, we performed fluoroscopy-guided nerve root blocks, injecting dexamethasone and mepivacaine. She was prescribed acetaminophen, pregabalin, and celecoxib (Figure 1).

**Figure 2:**
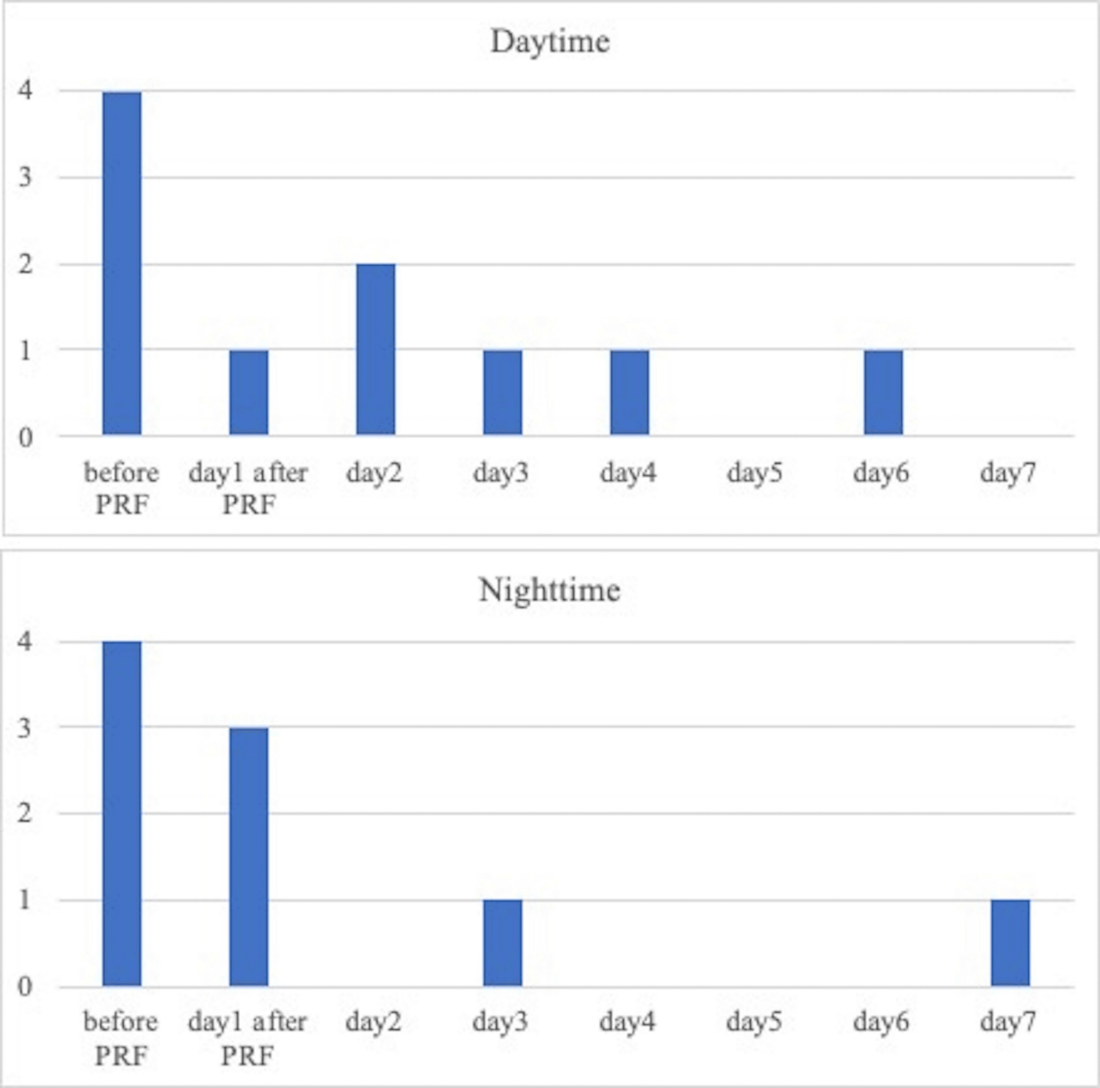
The change in Kawashima’s criteria of pruritus severity after PRF therapy PRF therapy was performed in the morning on day one PRF: pulsed radiofrequency

Although the throbbing pain got better, NRS was still 7. A second nerve root block was performed one week later. Two weeks post-visit, her pain improved but she developed severe itching (NRS: 8/10; Kawashima: 4/4). Itching was seen along the T6 nerve and she complained of an itch that would not go away, no matter how hard she scratched it. Given the neuropathic origin of her itching, we performed PRF therapy (parameters: 45 V, pulse temperature: 42 °C, pulse duration: 20 ms, pulse rate: 2 Hz, and pulse time 360 s) (Figure 2). The itching subsided to NRS 1/10 and Kawashima to 0/4 by the third week, and her medication was gradually tapered. At 120 days, a follow-up phone interview confirmed the absence of itching and pain.

## Discussion

PHI significantly affects the quality of life, often worsening at night time [1,2]. Unlike histamine-driven peripheral itching, PHI shares mechanisms with neuropathic pain, suggesting its neuropathic origin [3,7]. PHI is more common in females, often in the head and neck regions [1,8,9]. Treatments for PHI include medications, nerve blocks, and PRF therapy, but no standardized approach exists [2,4,6]. We used the NRS and Kawashima’s pruritus severity scale to assess the degree of itching and its impact on sleep [10,11]. In our clinic, we treat PHI similarly to neuropathic pain, using nerve root blocks and offering PRF or spinal cord stimulation if symptoms persist.

Concretely, we judged the severity of HZ by the number of risk factors for transition to PHI, such as the degree of skin lesions, the degree of pain, age, and other factors [12]. And if the risk is estimated as intermediate to high level, we perform nerve blocks [13]. We try nerve blocks two or three times initially, and if their effectiveness is limited to some extent, we perform PRF as soon as possible [14]. PRF is effective in treating neuropathic pain [5], is less invasive than other procedures, and has a low risk of complications, as it minimizes tissue destruction by limiting temperature elevation [15]. Cost-effectiveness studies also suggest that PRF may reduce the need for frequent outpatient visits [16]. This case report is limited to a single patient, and further studies are necessary to validate the generalizability of these findings.

## Conclusions

PRF therapy may be a viable treatment option for PHI, potentially expanding the range of therapeutic approaches available for this challenging condition. However, larger studies are needed to validate these findings and establish PRF as a standard treatment option for PHI.
